# Understanding the primary healthcare context in rural South and Southeast Asia: a village profiling study

**DOI:** 10.1093/inthealth/ihaf025

**Published:** 2025-03-20

**Authors:** Rusheng Chew, Sazid Ibna Zaman, Mst Asfat Ara Joly, Didar Uddin, Md Nurullah, James J Callery, Carlo Perrone, Thomas J Peto, Koukeo Phommasone, Aung Pyae Phyo, Wanlapa Roobsoong, Aninda Sen, Moul Vanna, Arjun Chandna, Tiengkham Pongvongsa, Lek Dysoley, Nicholas P J Day, Yoel Lubell, Richard J Maude

**Affiliations:** Mahidol Oxford Tropical Medicine Research Unit, 420/6 Ratchawithi Road, Ratchathewi, Bangkok 10400, Thailand; Centre for Tropical Medicine and Global Health, University of Oxford, New Richards Building, Roosevelt Drive, Headington Oxford OX3 7LG, UK; Faculty of Medicine, University of Queensland, Herston Road, Herston 4006, Brisbane, Australia; Mahidol Oxford Tropical Medicine Research Unit, 420/6 Ratchawithi Road, Ratchathewi, Bangkok 10400, Thailand; GroupMappers, 6/20 Block-E, Lalmatia, Mohammadpur, Dhaka 1207, Bangladesh; GroupMappers, 6/20 Block-E, Lalmatia, Mohammadpur, Dhaka 1207, Bangladesh; Mahidol Oxford Tropical Medicine Research Unit, 420/6 Ratchawithi Road, Ratchathewi, Bangkok 10400, Thailand; GroupMappers, 6/20 Block-E, Lalmatia, Mohammadpur, Dhaka 1207, Bangladesh; Mahidol Oxford Tropical Medicine Research Unit, 420/6 Ratchawithi Road, Ratchathewi, Bangkok 10400, Thailand; Centre for Tropical Medicine and Global Health, University of Oxford, New Richards Building, Roosevelt Drive, Headington Oxford OX3 7LG, UK; Mahidol Oxford Tropical Medicine Research Unit, 420/6 Ratchawithi Road, Ratchathewi, Bangkok 10400, Thailand; Chiang Rai Clinical Research Unit, 1038/8 Sanambin Road, Mueang Chiang Rai, Chiang Rai 57000, Thailand; Mahidol Oxford Tropical Medicine Research Unit, 420/6 Ratchawithi Road, Ratchathewi, Bangkok 10400, Thailand; Centre for Tropical Medicine and Global Health, University of Oxford, New Richards Building, Roosevelt Drive, Headington Oxford OX3 7LG, UK; Lao-Oxford-Mahosot Hospital-Wellcome Trust Research Unit, Quai Fa Ngum, Sisattanak, Vientiane, Laos; Shoklo Malaria Research Unit, 78/1 Moo 5, Mae Ramat, Tak 63140, Thailand; Mahidol Vivax Research Unit, Mahidol University, 420/6 Ratchawithi Road, Ratchathewi, Bangkok 10400, Thailand; BRAC, 75 Mohakhali, Dhaka 1212, Bangladesh; Action for Health Development, 91 Street 614, Rumchek 2, Sangkat Ratanak, Battambang 021403, Cambodia; Centre for Tropical Medicine and Global Health, University of Oxford, New Richards Building, Roosevelt Drive, Headington Oxford OX3 7LG, UK; Cambodia Oxford Medical Research Unit, Tep Vong and Um Chhay Street, Mondul 1, Svay Dangkum, Siem Reap 171202, Cambodia; Savannakhet Provincial Health Office, Khanthabouly Road, Kaysone Phomvihanh, Savannakhet, Laos; National Center for Parasitology, Entomology, and Malaria Control, 477 Betong Street, Trapaengsvay, Sangkat Phnom Penh Thmey, Phnom Penh 120801, Cambodia; Mahidol Oxford Tropical Medicine Research Unit, 420/6 Ratchawithi Road, Ratchathewi, Bangkok 10400, Thailand; Centre for Tropical Medicine and Global Health, University of Oxford, New Richards Building, Roosevelt Drive, Headington Oxford OX3 7LG, UK; Mahidol Oxford Tropical Medicine Research Unit, 420/6 Ratchawithi Road, Ratchathewi, Bangkok 10400, Thailand; Centre for Tropical Medicine and Global Health, University of Oxford, New Richards Building, Roosevelt Drive, Headington Oxford OX3 7LG, UK; Mahidol Oxford Tropical Medicine Research Unit, 420/6 Ratchawithi Road, Ratchathewi, Bangkok 10400, Thailand; Centre for Tropical Medicine and Global Health, University of Oxford, New Richards Building, Roosevelt Drive, Headington Oxford OX3 7LG, UK; Open University, Walton Hall, Kents Hill, Milton Keynes MK7 6AA, UK

**Keywords:** context, South Asia, Southeast Asia, village profiling

## Abstract

**Background:**

Understanding contextual factors is critical to the success of health service planning and implementation. However, few contextual data are available at the village level in rural South and Southeast Asia. This study addressed the gap by profiling representative villages across seven sites in Thailand (n=3), Cambodia, Laos, Myanmar and Bangladesh.

**Methods:**

Key informant surveys supplemented by other information sources were used to collect data from 687 villages on four key indicators (literacy rate, and percentages of attended deliveries, fully immunised children and latrine coverage), as well as access to various services. Data were analysed descriptively.

**Results:**

Sites varied considerably. Five were highly diverse ethno-culturally and linguistically, and all relied on primary health centres and village health/malaria workers as the main providers of primary healthcare. These were generally bypassed by severely ill patients for urban first-level referral hospitals and private sector facilities. While >75% of villages were near primary schools, educational attainment was generally low. Over 70% of villages at each site had mobile phone coverage and availability of electricity was high (≥65% at all sites bar Myanmar).

**Conclusion:**

These results illustrate the similarities and differences of villages in this region that must be considered in public health research and policymaking.

## Introduction

An understanding of contextual factors is crucial to the successful implementation of interventions to improve healthcare delivery. Although there is no one standard definition of ‘context’ as it relates to healthcare,^1^ one useful definition is the ‘situational opportunities and constraints that affect the occurrence and meaning of organizational behaviour as well as functional relationships between variables’,^2^ indicating that context necessarily includes both internal and external inter-related factors.^3^

In rural South and Southeast Asia, despite differences in health systems between countries in terms of financing and structure (Table 1), healthcare is principally delivered at the village, or village group, level through village health workers and primary health centres, with referral facilities typically being distant and relatively difficult to access.^4,5^ As such, interventions targeting primary healthcare would be expected to produce maximum impact. Nevertheless, as previous experience with primary health centres in India has shown, despite the apparent basing of health service provision on the principles of equity and administrative accountability, not catering to local contextual variations between villages and not involving local staff in service planning resulted in suboptimal healthcare delivery.^6^ The key variables identified relate to service coverage and socioeconomic and geographical factors,^6^ the determination of which have the benefit of being useful for micro-planning, that is, planning of health services and interventions at the village or subdistrict level.^7^

**Table 1.** Health system overview of SEACTN countries, with a particular focus on primary care service delivery.^10–14^ Universal health coverage, as defined by the WHO, means that all people have access to the full range of quality health services they need, when and where they need them, without financial hardship^15^

Country	Universal health coverage	Structure of primary healthcare system
Bangladesh	No (targeted for 2032)	• Delivered by public (under the Ministry of Health and Family Welfare and other ministries) and private sector providers, as well as NGOs • Public sector primary care is provided at all tiers of Ministry of Health and Family Welfare facilities from community PHC to tertiary hospitals; many villages have lay VHWs who can treat uncomplicated malaria and provide health promotion • PHC services include MNCH; reproductive health and family planning, nutrition and some communicable and non-communicable disease management
Cambodia	No (targeted for 2035)	• Delivered by public, private and non-medical sector providers • Public sector PHCs provide MNCH services, immunisation, nutritional education, family planning and some communicable and non-communicable disease management, as well as outreach to villages; at smaller, more remote communities >15 km from a PHC there may be a Health Post, and many villages have VHWs • PHCs are meant to be gatekeepers for secondary care, but in practice, patients are able to bypass them and go directly to public sector hospital outpatient clinics • Private providers deliver a major proportion of primary health care, often through government health staff working privately (dual practice)
Laos	No (targeted for 2025)	• Delivered by public and private sector (mainly in urban areas) providers • Rural villages have volunteer VHWs, who provide health education and some health promotion services • Public sector PHCs, each serving a catchment population of 1000–5000 people, provide treatment of common diseases, vaccinations, antenatal care, birth assistance, postnatal care and home visits to patients suffering from chronic conditions or with poor mobility
Myanmar*	No (targeted for 2030)	• Delivered by public and private sector providers • Public sector rural PHCs and sub-PHCs provide basic primary care services overseen by their respective township, district, and region and state health departments, which provide technical assistance and support
Thailand	Yes (excludes non-citizens)**	• Delivered by public and private sector providers • Each subdistrict (approximately 5000 people) has at least one public sector PHC that provides health promotion, prevention, medical treatment and rehabilitation services; malaria posts and clinics are also present in most malaria-endemic districts • Private clinics are widely available, and patients are able to bypass PHCs and go directly to public sector hospital outpatient clinics

Abbreviations: MNCH: maternal, neonatal and child health; NGO: non-governmental organisation; PHC, primary health centre; SEACTN: South and Southeast Asian Community-based Trials Network; VHW: village health worker.

*An armed conflict is currently occurring in large parts of Myanmar, with many areas not under the authority of the central government; this description only applies to areas under government control.

**SEACTN sites in Thailand are in border areas, where many non-Thai citizens not included in the national universal health coverage scheme access health services.

Constructing concise yet comprehensive village profiles that contain relevant key indicators is one way of characterising contextual factors important for the implementation of primary healthcare interventions and service planning. Such indicators must be as simple as possible, widely accepted and highly applicable to the village setting. They should also be easily comparable, sensitive to change and yield data that are meaningful and easy to analyse.^8^

While governmental authorities may have village-level data on some of these indicators, these may not be available from a single source and may not be current, especially as access to rural and remote locations is difficult. Correspondingly, there is also scant published research that aimed to depict the rural South and Southeast Asian primary healthcare context via the profiling of representative villages, let alone research that aimed to compare high-level village profiling data across countries. This study aimed to fill these knowledge gaps, primarily to understand better the findings of a large-scale observational study on the epidemiology of acute febrile illness in the region in which patients were recruited from the selected villages,^9^ but also to use the profiles to plan future research and guide policy-making.

## Methods

### Setting

**Figure 1.**
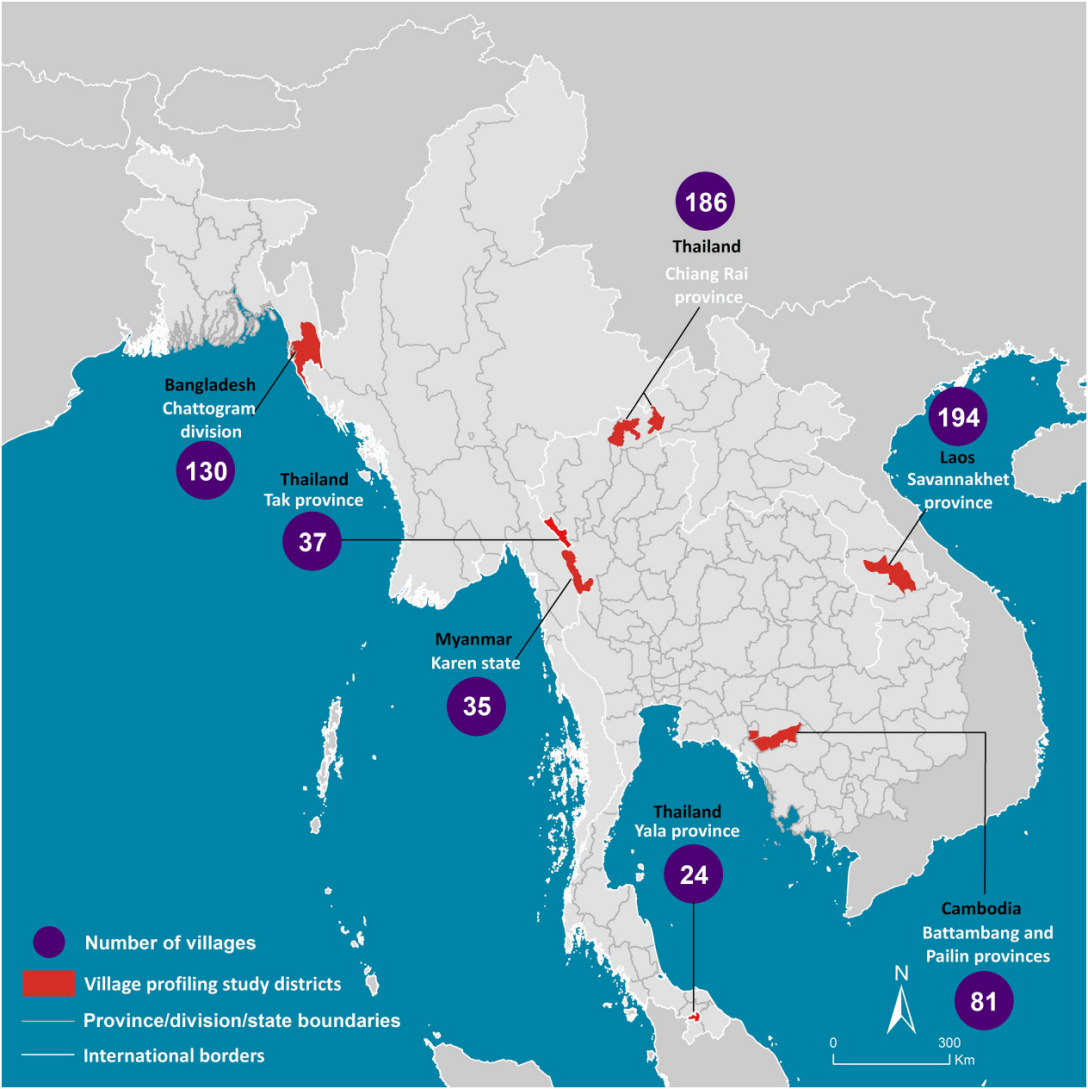
Locations of study sites in the South and Southeast Asian Community-based Trials Network and the number of villages profiled at each site.

This study was carried out across 687 of the 710 villages forming part of the South and Southeast Asian Community-based Trials Network (SEACTN), which spans seven sites across five countries (Thailand, Laos, Cambodia, Bangladesh and Myanmar) (Figure 1). The sites are located in rural districts of the following locations: Chiang Rai, Tak and Yala provinces, Thailand; Savannakhet province, Laos; Chattogram division, Bangladesh; Battambang and Pailin provinces, Cambodia; and Karen state, Myanmar. A full description of SEACTN is available in the open letter announcing its launch.^9^ Every village included in SEACTN was profiled, with the exception of 20 villages in Tak province, Thailand, and three villages in Karen state, Myanmar, which were not studied due to logistical difficulties and security concerns, respectively.

Participating villages were selected for inclusion in SEACTN by country implementing partners, who have the most knowledge to determine which villages are representative of the areas where the target populations for the observational study and other SEACTN project components reside, and where prospective, directly collected data would be most useful. Of note, one of the primary objectives of establishing SEACTN, which was to map and describe health services in underserved and understudied rural and remote areas of South and Southeast Asia, was a guiding principle in selecting villages.

An exception was made for the Myanmar site due to the ongoing armed conflict in Karen state,^16^ resulting in the need to reduce inaccessibility to ensure the viability of conducting other SEACTN activities. Therefore, while all villages profiled were rural, these were in the area near the Thai border rather than in the interior of Karen state, and better connected to transport and communication infrastructure. Because of this, and their borderland location, residents of these villages were also able to access facilities in Thailand, in particular health services.

### Study design

Similar to previous studies that collected data on key health indicators,^17^ we adopted a survey-based study design, with key informants used as respondents. These key informants were identified by site research staff, and included healthcare workers, village leaders and villagers who were judged to have the requisite knowledge. The number of key informants per village to interview was also determined by the country implementing partners, deferring to their knowledge on the prevailing conditions at each site and, thus, the most pragmatic and practicable approach to obtain the necessary information. Other data sources were used as relevant; these included Global Positioning System (GPS) data, census data and published government documents (e.g. official statistics). It should be noted, however, that a detailed exploration of health-seeking behaviour among residents of the study villages was beyond the scope of this study. This very important research will be the focus of other SEACTN projects; in fact, assessing health-seeking behaviour during acute febrile illness is an objective of the household health survey described in the open letter.^9^ This study will provide invaluable contextual detail to aid the interpretation of the results of these other projects.

### Questionnaire development

The survey questionnaire was developed iteratively through a series of discussions with senior researchers from each site. This approach was taken to ensure that only relevant and useful data were collected and, for questions where respondents were presented with options from which to select, the options were tailored to site-specific conditions.

The questionnaire consisted of four sections covering village location, socioeconomic conditions, health services and infrastructure, as well as public utilities. Questions on key health and socioeconomic indicators were adapted from the indicators of resources and service performance identified by Larson and Mercer as being well defined, valid, feasibly collected and useful, that is, literacy rate, percentage of attended deliveries, percentage of fully immunised children and percentage of latrine coverage.^8^

Given country-specific differences in the definition of literacy, a proxy measure of completion of at least 5 y of schooling in adults aged ≥18 y was used instead. As per the WHO, an attended birth was defined as one presided over by a skilled birth attendant (i.e. an accredited health professional), such as a midwife, doctor or nurse, who has been educated and trained to proficiency in the skills needed to manage normal uncomplicated pregnancies, childbirth and the immediate postnatal period, and in the identification, management and referral of women and neonates for complications. Traditional birth attendants, whether trained or not, are not classed as skilled birth attendants.^18^ For clarity, definitions of the various healthcare worker roles are shown in Table 2. A fully immunised child was defined as one who has received the following vaccines: Bacille Calmette-Guérin; three doses of diphtheria, pertussis and tetanus; three doses of polio; and measles by the age of 1 y.^8^ Estimates for educational attainment, skilled birth attendance and vaccine coverage were obtained by asking key informants to consider a representative sample of 20 persons relevant to the question, thus are rounded to the nearest 5% at the point of data collection.

**Table 2.** Definitions of healthcare worker roles used in this study. WHO definitions were used to define ‘auxiliary midwife’,^19^ ‘village health worker’,^20^ ‘traditional birth attendant’^21^ and ‘traditional healer’^22^

Role	Definition
Doctor	A healthcare worker who has completed a course of training in allopathic medicine at a medical school accredited or recognised in the country of practice
Nurse	A healthcare worker who has completed a course of training in nursing at a nursing school accredited or recognised in the country of practice
Pharmacist	A healthcare worker who has completed a course of training in pharmacy at a pharmacy school accredited or recognised in the country of practice
Primary health centre worker	A healthcare worker who is not a doctor, nurse or midwife and who has completed a course of training conducted by the local health authority to equip them to work in a paramedical capacity in a primary health centre
Auxiliary midwife	A healthcare worker who assists in the provision of maternal and newborn healthcare, particularly during childbirth, but also in the prenatal and postpartum periods. They may have had a period of on-the-job training, sometimes formalised in apprenticeships and possess some midwifery competencies but are not fully qualified as midwives. They also have basic nursing skills and no training in nursing decision-making
Village health worker	A layperson who has received some paramedical training (up to 2 y) but is not considered a health professional, and who is based in their village of residence (i.e. they provide services outside of health facilities or at peripheral facilities not staffed by health professionals)
Village malaria worker	A village health worker with a narrower remit focusing solely or primarily on the diagnosis and treatment of malaria in the community
Malaria post worker	Equivalent to a village malaria worker but who works in a malaria post
Traditional birth attendant	A layperson who assists a mother during childbirth and initially acquired their skills by delivering babies themselves, or through apprenticeship to other traditional birth attendants
Traditional healer	A layperson who has not had any formal medical training, but is considered by the local community as being competent to provide healthcare using animal, plant and mineral substances and certain other techniques based on social, cultural and religious background as well as the knowledge, attitudes and beliefs that are prevalent in the community regarding physical, mental and social well-being and the causation of disease and disability
Informal healthcare provider	A layperson who does not have any formal healthcare training, whether allopathic or traditional, but who claims to be competent to provide healthcare

The WHO/United Nations Children's Fund Joint Monitoring Program for Water Supply, Sanitation, and Hygiene (WHO/UNICEF JMP) defines ‘improved drinking water sources’ as those that are likely to be protected from outside contamination, particularly faeces. Examples include household connections, public standpipes, boreholes and protected dug wells.^23^ The WHO/UNICEF JMP also distinguishes between ‘improved’ and ‘unimproved sanitation facilities’, with the former defined as those that hygienically separate human waste from human contact including flush or pour-flush to piped sewer systems, septic tank pit latrines, ventilated-improved pit latrines or pit latrines with slab or composting toilets. Importantly, shared or public-use sanitation facilities are not considered to be improved.^23^ To describe access to health services and other relevant infrastructure and public utilities, key informants were asked to list the services available in the village or within a 30-min walk of their residence. This 30-min travel time was chosen as it has been widely used to define appropriate accessibility.^24,25^

The survey questionnaire template, which formed the basis for the study electronic data collection tool described in the next section, is shown in the Supplementary Data S1. Questionnaires for each site were translated into the main language used at that site to ensure that language was not a barrier.

### Data collection instrument and procedures

An electronic version of the survey questionnaire was developed using KoboToolbox (Kobo Organization, Cambridge, USA) and loaded onto tablet devices. Data collection was then carried out electronically for each village by site research staff using a combination of visits to the village, telephone interviews and desk-based research. Data collection at each site was conducted over a period of 1–2 mo. However, this was not carried out simultaneously at all sites as research staff were also engaged in other projects; the first site commenced data collection on 5 January 2023 and the last site to finish did so on 2 August 2024. Using the translated questionnaires as scripts, three to five local research staff at each site were tasked with data collection. These staff received appropriate training on conducting interviews and using the electronic data collection instrument by the relevant site study coordinator. Interviews were not recorded and transcribed, because the vast majority of the questions did not require free-text responses and could be answered either through selecting from a range of options or by providing a short response in the form of a number.

Village population figures were obtained from the latest available official statistics; for villages where these were not available, estimates were obtained through key informant interviews.

Key informants were interviewed either in person or by telephone. At sites where more than one key informant was interviewed per village, the mean of the responses to questions requiring numerical answers was entered as the final value if the responses were broadly similar. Otherwise, research staff were allowed to use their discretion in selecting the preferred response based on how well-suited the knowledge and expertise of the key informant was to the question, for example, the response of a health worker key informant would be preferred over that of a village head for the question on percentage of attended deliveries.

### Statistical analysis

Data were analysed and presented using descriptive statistics to depict an ‘average’ village for each site. Count data at the site level were reported directly, while medians and upper and lower quartiles were calculated for continuous data captured at the village level. Percentages were calculated for binary and categorical data captured at the village level.

Responses to questions requiring the selection of one or more responses (e.g. types of water source available at each village) were summarised graphically to show the proportion of villages per site with access to a particular service. Analyses were carried out using Excel (Microsoft, Redmond, USA).

## Results

**Figure 2.**
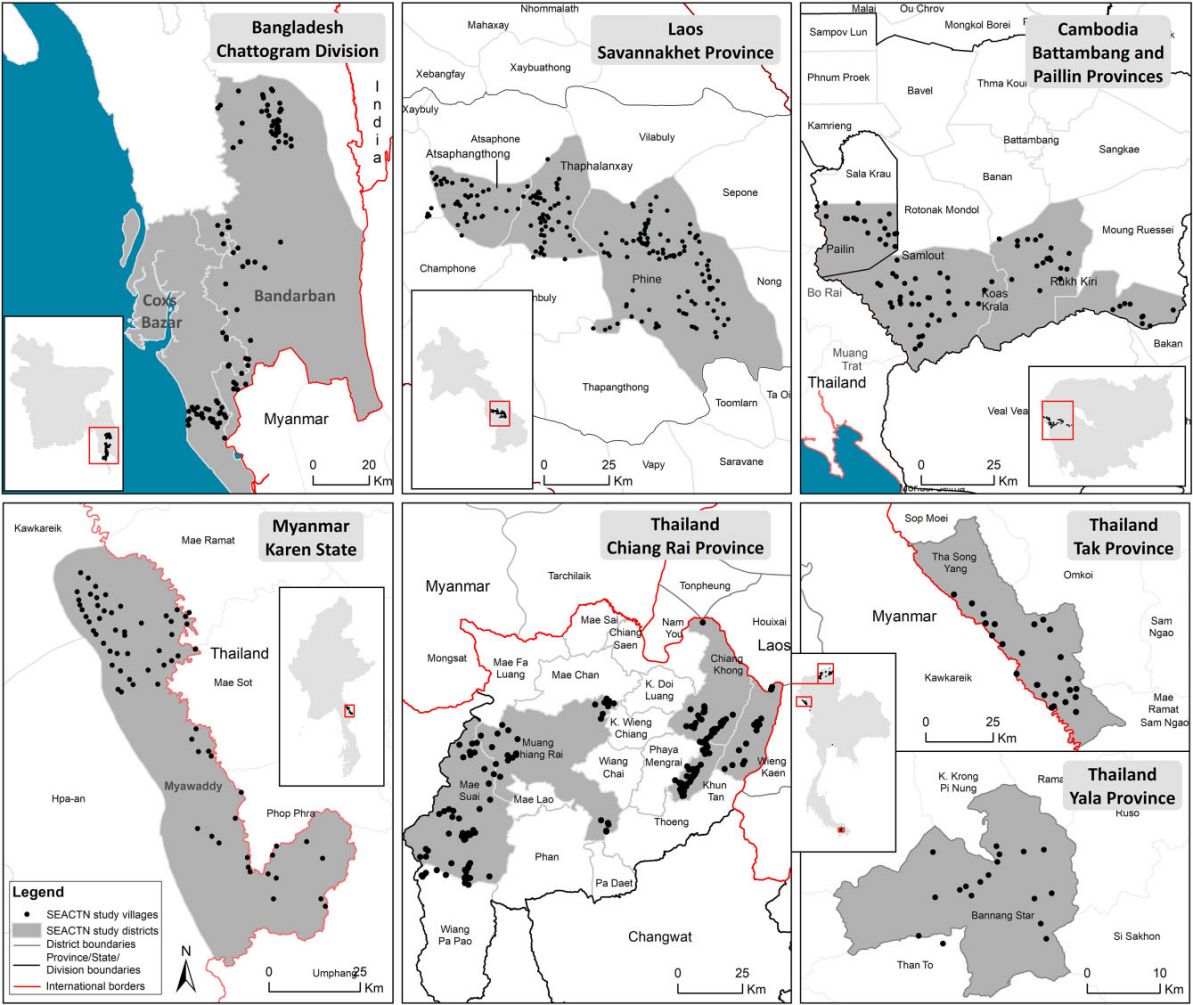
Locations of profiled villages within each study site of the South and Southeast Asian Community-based Trials Network.

The locations of the villages profiled in this study within each study site are shown in Figure 2.

### Demographic details and health and socioeconomic status indicators

Site-specific demographic and health and socioeconomic status indicator data are shown in Table 3.

**Table 3.** Demographic and key health and socioeconomic indicator data for rural villages in the South and Southeast Asian Community-based Trials Network by site. Estimates are presented as medians (first quartile, third quartile) and n (%) unless otherwise indicated

Country	Thailand			Laos	Cambodia	Myanmar	Bangladesh
Site (number of villages profiled)	Chiang Rai province (186)	Tak province (37)	Yala province (24)	Savannakhet province (194)	Battambang and Pailin provinces (81)	Karen state (35)	Chattogram division (130)
Number of key informants per village, n	1 (Village head or PHC staff member)	1 (VHW/VMW)	1 (VHW/VMW)	1 (Village head)	1 (Village head)	2 (Village head and VHW/VMW)	2–3 (Village head and VHW/VMW)
*Demographic details*							
Population per village, n	555 (422, 769)	902 (501, 1245)	345 (238, 621)	750 (486, 1135)	639 (417, 1127)	676 (340, 1021)	526 (240, 1366)
Households per village, n	180 (129, 234)	240 (122, 372)	101 (78, 147)	ND	169 (100, 275)	129 (64, 215)	121 (57, 267)
Main languages at site, n	10	4	2	4	1	8	8
Main religions at site, n	3	4	2	3	2	6	3
*Health and socioeconomic indicators*							
Number of villages with a health facility within a 30-min walk (%)	105 (56.5)	35 (94.6)	15 (62.5)	55 (28.4)	61 (75.3)	35 (100)	114 (87.7)
Longest travel time to closest health facility by motor vehicle if none within 30-min walk (min)	60	30	15	ND	90	NA	80
% adults with ≥5 y schooling* Male Female	75 (65, 100) 75 (60, 90) 70 (55, 80)	60 (50, 85) 50 (50, 90) 60 (50, 90)	60 (50, 70) 50 (45, 55) 70 (65, 75)	ND	100 (90, 100) 100 (75, 100) 100 (55, 100)	50 (70, 75) 60 (40, 70) 70 (55, 80)	70 (60, 80) 65 (50, 75) 65 (45, 80)
% attended deliveries^§^	100 (100, 100)	10 (5, 27.5)	ND	ND	100 (100, 100)	80 (47.5, 95)	60 (25, 85)
% fully immunised children^†^	100 (100, 100)	100 (95, 100)	67.5 (58.8, 75)	ND	100 (100, 100)	75 (85, 95)	100 (100, 100)
% latrine coverage^‡^	97.3	83.1	92.3	ND	86.7	84.8	67.5

NA: not applicable; ND: data not collected or not available due to local restrictions, particularly in Laos; PHC: primary health centre; VHW/VMW: village health worker/village malaria worker.

*Completion of at least 5 y of schooling was used as an indicator of literacy given varying definitions of literacy between countries.

^§^A delivery attended by a skilled birth attendant.

^†^A child aged >1 y who has received the following vaccines: Bacille Calmette–Guérin; three doses of diphtheria, tetanus and pertussis; three doses of measles, and polio.

^‡^Proportion of households across all villages at each site that have either flush toilets or latrines.

In the profiled SEACTN villages, the median village population for any given site ranged from 345 to 902 people. There was a high level of cultural, religious and linguistic diversity with several languages being spoken and religions practised at all sites, except in Cambodia and Yala province of Thailand, which were more homogenous.

In general, the median percentage of adults with 5 y of schooling was no greater than 75%, except in Cambodia where this figure was 100%. There was little difference in educational attainment between genders, except in Yala province, Thailand, where a median of 70% of females had achieved this level of education compared with 50% of males.

Except for Chiang Rai and Yala provinces in Thailand, latrine coverage did not exceed the 90% threshold generally accepted as the coverage required to have a positive impact on the health of the community.^26^ Latrine coverage was particularly low in Bangladesh at 67.5%.

Vaccination coverage was low in Myanmar and Yala (median coverage of 75% and 67.5%, respectively) relative to other sites that had near-perfect coverage.

### Access to health services

The percentage of villages with access to a health facility within a 30 min-walk was highly variable, ranging from 28.4% in Laos to 100% in Myanmar. The travel times to the nearest health facility for villages with no facility within a 30 min-walk ranged from 15 to 90 min by motor vehicle. The median percentage of deliveries attended by skilled birth attendants was also markedly lower in Bangladesh (60%) compared with other sites.

**Figure 3.**
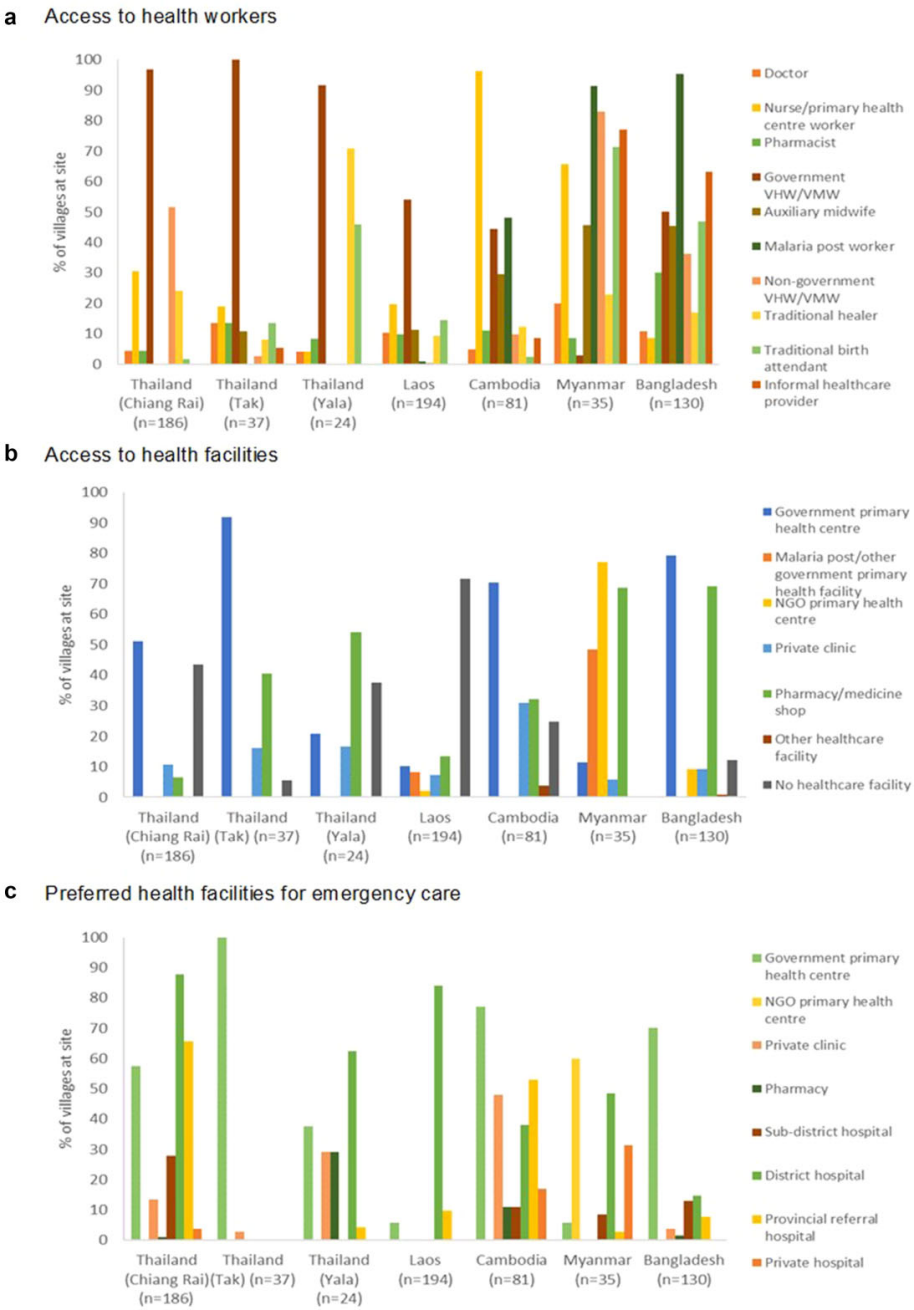
Percentages of villages in South and Southeast Asian Community-based Trials Network Rural Febrile Illness project study sites with access to different types of (a) healthcare worker and (b) healthcare facility. Percentages of villages and the types of healthcare facilities at which patients perceived as being severely ill from those villages would seek care are shown in (c). Access was defined as having the healthcare worker or facility in the village or within a 30-min walk. NGO: non-governmental organisation; VHW/VMW: village health worker/village malaria worker.

As might be expected, few villages at all sites had access to doctors; the percentage of villages that had a doctor on site or within a 30-min walk ranged from 4.2% in Yala to 20.0% in Myanmar (Figure 3a). By far the commonest types of health worker in the studied villages were primary health centre (PHC) workers/nurses and village health workers/village malaria workers (VHWs/VMWs). Interestingly, traditional healers were not found in most villages across all sites, with the exception of Yala, where 70.8% of villages have them. All villages had access to a healthcare worker. Nevertheless, it should be borne in mind that many healthcare providers are informal, with little or no official training. These informal healthcare providers were found in 63.1% and 77.1% of villages in Bangladesh and Myanmar, respectively, and do not include traditional healers or traditional birth attendants; however, in all of the villages where they were present, there was also at least one type of formal healthcare provider.

It should be of little surprise given the above findings that most facility-based healthcare in the profiled villages was delivered at PHCs (Figure 3b), and that where they are not, such as in Yala and Laos where there are few PHCs, the burden of primary healthcare delivery falls on VHWs/VMWs. This is most important in Laos, where 71.6% of villages had no healthcare facility, including pharmacies or medicine shops, within a 30-min walk. Notably, also, in Myanmar, 77.1% of villages were served by clinics set up by non-governmental organisations (NGOs) vs 11.4% served by government PHCs. It is also important to note the relatively easy access to pharmacies or medicine shops at all sites except Chiang Rai and Laos. The degree to which these businesses are regulated varies between countries, and even within countries there may be a divergence between theory and practice. On the whole, however, regulation of pharmacies and medicine shops is loose, with medicines generally available over the counter.

As shown also in Figure 3b, there are no secondary care facilities able to care for severely ill patients within a 30-min walk of any profiled village, with all easily accessible healthcare facilities being designed for the provision of basic primary care and/or diagnosis and treatment of uncomplicated malaria, in the case of malaria posts. It is, therefore, not unexpected that patients who perceive themselves as being severely unwell would generally primarily seek care from first-level referral hospitals, that is, those at the subdistrict and district levels, with the exception of Yala, Cambodia and Bangladesh (Figure 3c). At these three sites, the commonest facility to which severely unwell patients would present remains the PHC; this was a preferred option for emergency care in 37.5%, 77% and 70% of villages, respectively. However, the large role of private sector facilities, such as private hospitals and clinics, in treating severely ill patients in Cambodia, Myanmar and Yala should also be noted; at these sites, such facilities are a preferred option for emergency care in 65.0%, 31.4% and 29.2% of villages, respectively.

### Access to public utilities and education

#### Water and sanitation

**Figure 4.**
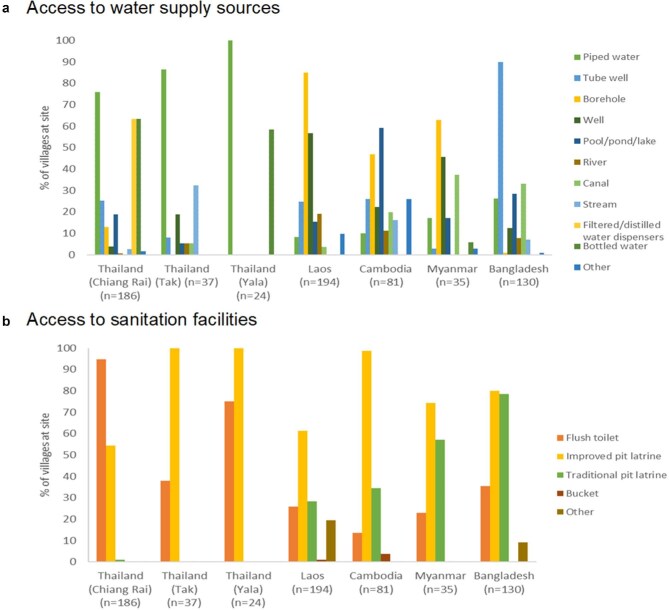
Percentages of villages in South and Southeast Asian Community-based Trials Network Rural Febrile Illness project study sites with access to different types of (a) water supply sources and (b) sanitation facilities. Access was defined as having the water source or sanitation facility in the village or within a 30-min walk.

Thai villages had the best access to improved drinking water sources, with the majority of villages having access to piped water available for at least most of the day (Table 4). Additionally, more than one-half of the villages studied in the Thai provinces of Chiang Rai and Yala were able to obtain bottled water and/or filtered or distilled water via dispensers; by contrast, <10% of Cambodian and Lao villages were supplied with piped water, with the principal water sources being wells and boreholes (Figure 4a). Overall, however, a higher percentage of villages had access to improved rather than unimproved drinking water sources, and the majority of villages without piped water had their water sources located within the village (Figure 4a).

**Table 4.** Access to public utilities and education for rural villages in the South and Southeast Asian Community-based Trials Network by site. The number of hours per day piped water and mains power were available, and distance to nearest school if not within a 30-min walk, are presented as median (first quartile, third quartile)

Country	Thailand			Laos	Cambodia	Myanmar	Bangladesh
Site (number of villages profiled)	Chiang Rai province (186)	Tak province (37)	Yala province (24)	Savannakhet province (194)	Battambang and Pailin provinces (81)	Karen state (35)	Chattogram division (130)
*Access to public utilities*							
Number of villages with water available year-round (%)	150 (80.6)	32 (86.5)	24 (100)	ND	55 (67.9)	22 (62.9)	128 (98.5)
Number of villages with piped water (%)	141 (75.8)	32 (86.5)	24 (100)	16 (8.2)	8 (9.9)	29 (82.9)	34 (26.2)
Hours/day piped water available*	24 (24, 24)	24 (24, 24)	15 (13, 16)	ND	24 (24, 24)	24 (12, 24)	20 (18, 24)
Number of villages without piped water but with intra-village water source (%)	40 (21.5)	3 (8.1)	NA	ND	65 (80.2)	4 (11.4)	95 (73.1)
Number of villages with mains power (%)	179 (96.2)	24 (64.9)	24 (100)	ND	63 (77.8)	2 (5.7)	115 (88.5)
Hours/day mains power available^§^	24 (24, 24)	24 (18, 24)	24 (24, 24)	ND	24 (24, 24)	6 (3.5, 15)	18 (17.5, 20)
Number of villages with mobile phone signal (%)^†^	186 (100)	26 (70.3)	20 (83.3)	194 (97.8)	81 (100)	35 (100)	128 (98.5)
*Access to education*							
Number of villages with a school within a 30-min walk (%)	177 (95.2)	37 (100)	20 (83.3)	181 (93.2)	62 (76.5)	32 (91.4)	125 (96.2)
Distance to nearest school if not within a 30-min walk, km	4.0 (2.0, 9.0)	NA	12.5 (9.3, 15.0)	4.0 (2.0, 5.0)	5.0 (2.9, 10.3)	3.3 (2.6, 6.7)	5.0 (4.0, 6.0)

NA: not applicable; ND: data not collected or available due to local restrictions, particularly in Laos.

*For villages with piped water.

^§^For villages with mains power.

^†^Defined as mobile phone signal present at least some of the time.

Unsurprisingly, the quality of sanitation facilities at each site mirrored the availability of piped water, with the percentage of villages with access to flush toilets being the highest in Chiang Rai (94.6%), and the lowest in Cambodia (13.6%) (Figure 4b). Overall, most villages relied on improved pit latrines (Figure 4b). However, data on whether these were shared/public vs private were not collected, although it is likely that at least some were shared facilities, especially in Bangladesh where 6.1% of villages were noted to have non-functioning latrines.

#### Electricity and light sources, and mobile phone coverage

**Figure 5.**
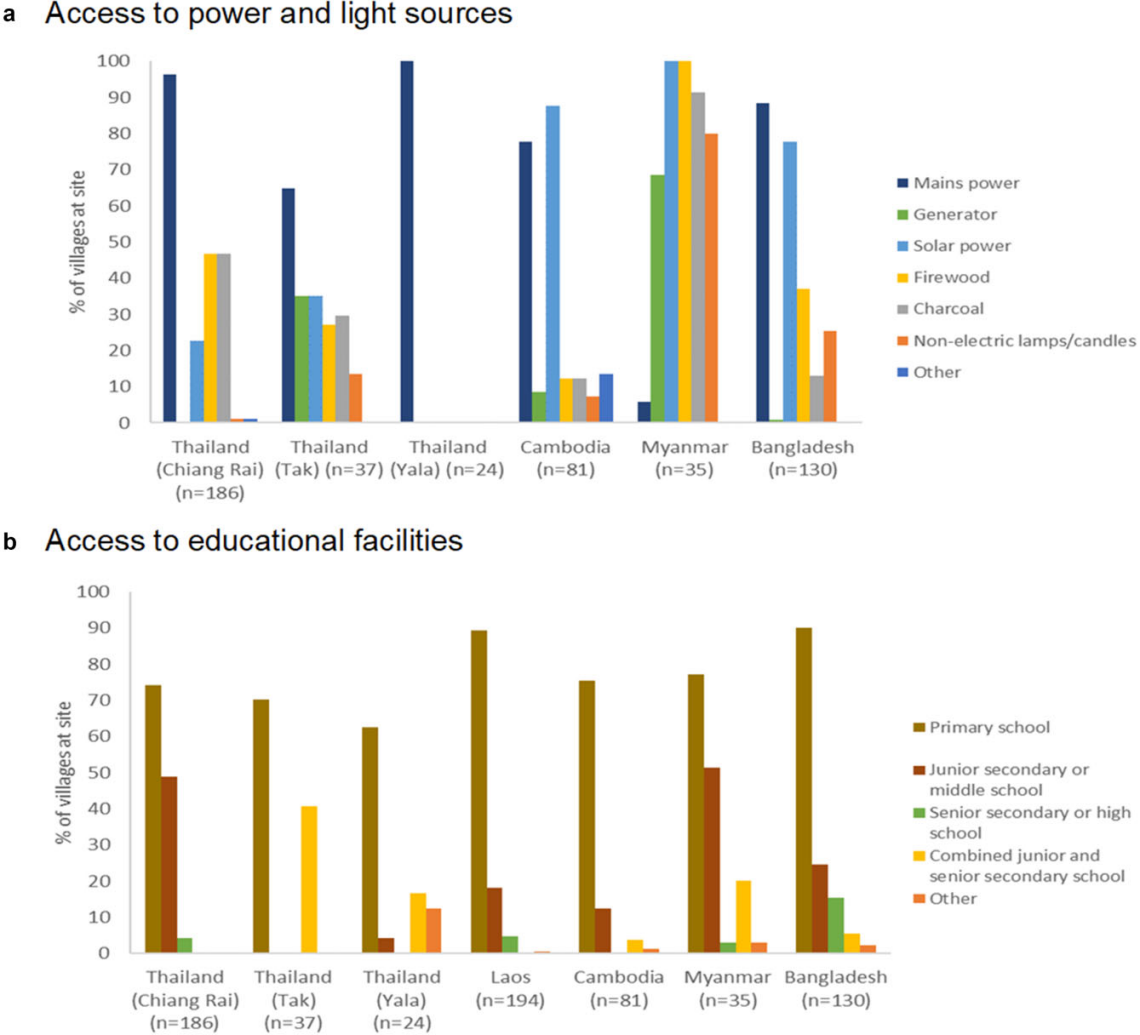
Percentages of villages in South and Southeast Asian Community-based Trials Network Rural Febrile Illness project study sites with access to different types of (a) power and light sources and (b) educational facilities. Access to power and light sources was defined as having the power and light source in the village, while access to educational facilities was defined as having the facility in the village or within a 30-min walk.

With the exception of Myanmar, >60% of villages at each site had access to mains electricity, with the highest penetration being in Yala (100%). Only 5.2% of villages at the Myanmar site were supplied with mains power; at this site, electricity was mainly provided by via generators or solar panels (Figure 5a). However, even in villages with mains power, supply was not always reliable, with the median number of hours per day ranging from six in Myanmar to 24 in Thailand (Table 4).

Regardless of source, electricity was generally available at the profiled villages even if not all households had reliable and affordable access to it, as evident by the continued use of firewood, charcoal and candles (Figure 5a). The availability of electricity supports the use of mobile phones, evidenced by the widespread signal coverage across all sites (Table 4).

#### Educational facilities

Across all sites, the majority of villages had a primary school within a 30-min walk (Figure 5b), with an even higher percentage having any type of educational facility within this distance (Table 2). The site with the lowest access to educational facilities was Yala, where 62.5% of villages had a primary school within a 30-min walk (vs 90% in Bangladesh). Furthermore, for villages in Yala with no school within this travel time, the median distance to the nearest school was longest compared with similar villages at all sites at 12.5 km (vs ≤5 km at the other sites) (Table 4).

## Discussion

This study profiled 687 villages located in five South and Southeast Asian low- and middle-income countries at different stages of development, revealing very different characteristics, especially in terms of access to services and public utilities. In line with this, therefore, there was considerable variation between sites in terms of health and socioeconomic indicators.

Nevertheless, several common themes were evident, the first being the obvious ethno-cultural and linguistic diversity that exists even within villages at the same site. While health-seeking behaviour was not explored in this study, the influence of such diversity should be investigated as part of future work on this very important issue. The second is their reliance on PHCs (and analogous NGO primary health clinics), and VHWs/VMWs, as the backbone of primary healthcare provision. The latter are particularly common in Thailand, Laos, Bangladesh and Cambodia where they have been a longstanding feature of village healthcare over the past decades. However, severely ill patients would generally travel out of their villages to seek care at first-level referral hospitals and private sector facilities in towns, bypassing village-level health services. Third, while most villages were proximate to a primary school, the level of education was generally low. Fourth, mobile phone coverage and availability of electricity was generally high.

Several findings require further explanation. First, the low vaccine coverage in Myanmar and Yala may partially be explained by these locations being conflict zones,^16,27^ with an additional layer of mistrust of the government and, by extension, government programmes by the majority Malay ethnic group in Yala.^27^ Furthermore, the low coverage of villages by public sector PHCs in Myanmar is due to the traditionally meagre government expenditure on healthcare,^28^ encouraging a parallel system of primary health clinics set up by NGOs to meet demand. This is especially so in Karen State where control by the central government has been patchy.^29^ Lastly, while not all villages were supplied by electricity through power grids and, even in those that were, the number of hours per day that such supply is available is limited, yet mobile phone coverage was high across all sites. This is due to innovative solutions having been devised to overcome the lack of private household electricity supply, such as grocery shops installing shared solar-powered mobile phone charging points in villages in Myanmar.^30^

The principal strength of this study is its focus on key health and socioeconomic indicators, data on which public health and other policymakers, as well as researchers, can use for planning purposes. Furthermore, a pragmatic, low-cost, semidesk-based methodology using key informants was chosen. This approach allowed reasonable estimates of the key indicators in the absence of easily accessible official information for all domains, in addition to the difficulties posed by the remote locations of many villages and travel restrictions for security reasons to some (e.g. those in the Chattogram Hill Tracts in Bangladesh). It also has the benefit of allowing high-quality spatially granular data to be collected, permitting detailed comparisons to be made. The key informants were selected by staff of SEACTN country partner organisations to ensure a high degree of first-hand knowledge of the prevailing conditions in their respective villages, as is best practice.^31^ This approach is well-suited to the objectives of this study, as it is appropriate if general, descriptive information is sufficient to guide decision-making, generate suggestions and recommendations, and assist in the design of more comprehensive quantitative research.^32^ Key informant interviews are also appropriate for when quantitative data collected through other methods need to be interpreted,^32^ which is exactly the situation with SEACTN where the data from the observational epidemiological studies being conducted at these sites need to be viewed through a contextual prism.^33^

Nevertheless, while the results provide an idea of the characteristics of villages at the individual sites, they may not be generalisable to all rural villages within their respective countries. This is amply demonstrated by heterogeneity of the three Thai sites, despite all being located in remote areas bordering neighbouring countries; there are likely to be many more differences between these borderland villages and those in the more developed central plains. In the case of Cambodia, the relative ethno-linguistic homogeneity and high elementary educational attainment of the villages profiled for this study would not likely be found in villages in the northeast of the country bordering Laos, where more languages are spoken and literacy rates are lower.^34^ The selected villages at the Myanmar site had better road and telecommunication links than others due to the logistical and security considerations previously described. This is likely to have resulted in better access to water and sanitation, health services and educational facilities, which may not be representative of the typical conditions elsewhere in Karen state or Myanmar in general. For example, there is an international hospital in Shwe Kokko close to several villages profiled at the Myanmar site, whereas no such facilities exist deeper in the interior of Karen state. Also, the higher immunisation coverage in these villages compared with the very low or negligible figures reported for villages elsewhere in Karen state^35^ is likely due to the ability of residents to access the more advanced health services in Thailand, including preventative care, as mentioned earlier. While it would have been useful to obtain data on employment status and median incomes at each village, it would have been difficult to obtain reliable estimates and compare these across sites without also assessing relative purchasing power, which the study was not resourced to perform.

Additionally, there are several well-known limitations of the key informant interview approach that should be borne in mind when interpreting the results of this study. Chief of these is the potential for bias because it was not possible to confirm the appropriateness of informant selection; for example, more qualified but less visible or prominent informants may not have been selected.^36^ It is also possible that, due to their prominence, the sociodemographic strata from which key informants are drawn may not be similar to those of the majority of village dwellers, in particular those from the most marginalised groups, leading to a perhaps less than comprehensive understanding of their villages. This should be taken into account when interpreting the results, because their potentially differing perspectives may have caused them to overestimate their responses to questions on health and socioeconomic indicators that require numerical answers. It is particularly important to consider this effect at the Myanmar site because, added to that of the relatively better access to services of the profiled villages at this site, it may have resulted in results that are less representative of more typical villages there. This notwithstanding, given that most of the data gathered related to demographic makeup and availability and accessibility of physical facilities such as schools, public utilities and healthcare services at each village, it would be quite unlikely that highly divergent answers would be provided for these questions because it was expected that this would be common knowledge to most, if not all, adult villagers, and certainly to the village head and healthcare workers servicing the village.

Another source of bias that should be considered is interviewer bias, although this was mitigated by using a data collection tool with questions to be read out verbatim by the interviewer, as well as having many questions with response options or which required numerical responses. However, the questions that required quantitative responses were in themselves a weakness, because key informant interviews are best suited to qualitative studies because they provide only a very restricted basis for quantification.^32^ It is also difficult to verify the validity of the findings because only a very small number of informants were selected per village, certainly fewer than the ideal figure of 15–35,^36^ which was deemed to be impracticable by all partner organisations. Hence, while most sites interviewed one per village and two interviewed two to three, this difference was inconsequential. Additionally, because the results shown in this report are intended to be a high-level overview and a large number of villages were included at each site, a not unreasonable assumption would be that the measures of central tendency presented would converge to a decently close approximation of the relevant true value for each site, as per the ‘wisdom of the crowd’ theorem.^37,38^ A final limitation of this study is that it while it may indicate the presence or absence of particular facilities or services, it gives no indication of their quality.^39^

There are two obvious priorities for further research. The first is to flesh out the findings with the results of other studies examining broadly similar themes, such as the SEACTN household health survey.^40^ In addition, more detailed site-specific analyses triangulating the findings of this study with geospatial epidemiological work analysing accessibility to health and other services will be extremely informative. Such efforts will allow further layers of contextual understanding to be built on the foundations established by this study. Second, this study has uncovered the major public health and socioeconomic issues important to each site, for example, the low vaccination rates in Yala province, the low percentage of births attended by skilled attendants in Bangladesh and the globally poor levels of educational attainment despite relatively easy physical access to primary schools. While this paper presents high-level overviews of these problems, the granular, village-level data collected through this study can be leveraged for the purposes of micro-planning and further research, among others. Future studies should explore the reasons behind such problems in the light of the other contextual findings, with a view to developing potential interventions to solve these issues.

### Conclusion

This study provides a rudimentary descriptive overview of the characteristics of underserved and understudied villages in rural South and Southeast Asia, focusing on key health and socioeconomic indicators and the availability and accessibility of amenities and public services essential to good health, such as water and sanitation. Notwithstanding its weaknesses, the results illustrate the wide diversity in the region and this should be considered in public health research and policymaking.

## Supplementary Material

ihaf025_Supplemental_File

## Data Availability

All data relevant to the study are included in the article or available upon reasonable request from the corresponding author.
