# Supplementary material for: Understanding the primary healthcare context in rural South and Southeast Asia: a village profiling study
Source: Int Health. 2025 Mar 20;17(5):754–68. doi: 10.1093/inthealth/ihaf025 (PMC12406796; doi:10.1093/inthealth/ihaf025)
Supplement: ihaf025_Supplemental_File [file ihaf025_supplemental_file.docx]

**Questionnaire for Village Profiling – what we would like to know for each village**

**Section 1: Location**

| **Item** | **Question** | **Response** |
| --- | --- | --- |
| 1.1 | Village name (if more than one name exists, enter the official name first followed by any other names, separated by “/”) |  |
| 1.2 | Village code (government) (where available) |  |
| 1.3 | Village code (SEACTN) |  |
| 1.4 | Sub-district/upazila/township name |  |
| 1.5 | Sub-district/upazila/township code |  |
| 1.6 | District name |  |
| 1.7 | District code |  |
| 1.8 | Province/division/state name |  |
| 1.9 | Province/division/state name |  |

**Section 2:** **Socio-economic Conditions** [*To be collected over phone or visit villages, best to talk to other programmes professional, census officials, local authorities or villagers*]

| **Item** | **Question** | **Response** |
| --- | --- | --- |
| 2.1.1 | What is the total population in the village? | ________________ people |
| 2.1.2 | What is the source of this information? | **□** Official census  **□** Headcount  **□** Personal knowledge estimate  **□** Other – please specify ________________________ |
| 2.1.3 | If not a personal knowledge estimate, when was this data collected (year)? | **□** Year ________________  **□** Not known |
| 2.1.4 | To your knowledge, how many households are there in the village? | ________________ households |
| 2.2.1 | What is the majority ethnic group in the village (select one option)? | **□** [Site-specific options]  **□** Other – please specify ________________________  **□** No dominant ethnicity |
| 2.2.2 | Which languages are used by the people of the village (select all that apply)? | **□** [Site-specific options]  **□** Other – please specify ________________________ |
| 2.2.3 | What are the main religions practiced in the village (select all that apply)? | **□** Buddhism  **□** Christianity  **□** Hinduism  **□** Islam  **□** Animism or traditional folk religion  **□** Other – please specify ________________________ |
| 2.3.1 | If you randomly picked 20 adults* (of any gender) in this village, how many would have finished 5 years of schooling?  **An adult is a person aged 18 years and above* | ________________ people |
| 2.3.2 | What if you only picked 20 male adults in this village, how many would have finished 5 years of schooling? | ________________ males |
| 2.3.3 | What if you only picked 20 female adults in this village, how many would have finished 5 years of schooling? | ________________ females |

**Section 3:** **Health services and infrastructure**

| **Item** | **Question** | **Response** |
| --- | --- | --- |
| 3.1 | What are the existing health services available in the village or within 30 minutes’ walk (select all that apply)? | **□** Government primary health centre  **□** Other government primary health facility – please specify _______________  **□** Non-government health centre  **□** Other non-government primary health facility – please specify _______________  **□** Private clinic  **□** Pharmacy  **□** Other shop selling medicines  **□** Other – please specify ________________________  **□** None |
| 3.1.1 | (Only show this question if the answer to 3.1 is ‘None’) What is the type of health facility closest to the village (select one only) and how long is the travel time to this facility? | **□** Government primary health centre  **□** Other government primary health facility – please specify _______________  **□** Non-government health centre  **□** Other non-government primary health facility – please specify _______________  **□** Private clinic  **□** Pharmacy  **□** Other shop selling medicines  **□** Other – please specify ________________________  Travel time ____________ minutes by **□** foot **□** motor vehicle |
| 3.2 | Which types of healthcare worker are providing health services in the village (select all that apply)? | **□** Medical doctor  **□** Nurse  **□** Pharmacist  **□** Government village/community health worker/healthcare assistant  **□** Non-government village/community health worker  **□** Traditional healer  **□** Traditional birth attendant  **□** Auxiliary midwife  **□** Malaria post worker  **□** Informal healthcare provider  **□** Other – please specify ________________________ |
| 3.3 | Where do seriously ill people first go for treatment (full name and type of health facility, select all that apply)? | **□** Government primary health centre  **□** Other government primary health facility – please specify _______________  **□** Non-government primary health centre  **□** Other non-government primary health facility – please specify _______________  **□** Private clinic  **□** Pharmacy  **□** Other shop selling medicines  **□** Sub-district/upazila/township hospital  **□** District hospital  **□** Provincial/divisional/state referral hospital  **□** Private hospital  **□** Other – please specify ________________________  **□** None |
| 3.4 | Consider the last 20 women from this village who have given birth. How many had the delivery conducted by a skilled birth attendant*?  **A skilled birth attendant is an accredited health professional - such as a midwife, doctor or nurse - who has been educated and trained to proficiency in the skills needed to manage normal (i.e. uncomplicated) pregnancies, childbirth and the immediate postnatal period, and in the identification, management and referral of women and neonates for complications. Traditional birth attendants, whether trained or not, are not skilled birth attendants.* | ________________ women |
| 3.5 | Consider 20 children in this village older than 1 year old. How many have had all the following vaccines?   - BCG - 3 doses of DTP - 3 doses of measles - Polio | ________________ children |

**Section 4:** **Public Utilities** [*To be collected over phone or visit villages, best to talk to local authorities or villagers*]

| **Item** | **Question** | **Response** |
| --- | --- | --- |
| 4.1.1 | What are the main sources of drinking water for the village (select all that apply)? | **□** Piped water  **□** Tube well  **□** Borehole  **□** Well  **□** Pool/pond/lake  **□** River  **□** Canal  **□** Stream  **□** Other – please specify ________________________ |
| 4.1.2 | If piped water is available, for how many hours each day on average? | ________________________ hours |
| 4.1.3 | If piped water is not available to all households, where is the main waterpoint located? | **□** In the village  **□** Outside the village |
| 4.1.4 | If outside the village, estimate approximate travel time to the waterpoint. | _______________________ minutes |
| 4.1.5 | Is water available throughout the year in the village? | **□** Yes  **□** No |
| 4.2.1 | What methods of communication are available in the village (select all that apply)? | □ Radio  □ Television  □ Landline phone  □ Mobile phone  □ Internet  □ Post office  □ Other |
| 4.2.2 | (Only show this question if ‘Mobile phone’ is selected in 4.2.1) Is there mobile phone signal in the village? | **□** Yes, all of the time  **□** Yes, some of the time  **□** No |
| 4.3.1 | What type of energy supply is available in the village (select all that apply)? | **□** Mains power (national or international source)  **□** Generator  **□** Solar power  **□** Firewood  **□** Charcoal  **□** Non-electric lamps/candles  **□** Other – please specify ________________________ |
| 4.3.2 | If mains power is available, for how long each day on average? | ________________________ hours |
| 4.4.1 | What types of toilet are available in the village? | **□** Flush  **□** Improved pit latrine  **□** Traditional pit latrine  **□** Bucket  **□** Other – please specify ________________________ |
| 4.4.2 | The number of households in this village was earlier estimated at [number]. How many of these households have either flush toilets or latrines? | ____________________ households |
| 4.5.1 | Is there a school(s) in the village or within 30 minutes’ walk? | **□** Yes  **□** No |
| 4.5.2 | If yes, type of school(s) (select all that apply). | **□** Primary school  **□** Junior secondary or middle school  **□** Senior secondary or high school  **□** Combined junior and senior secondary (high) school  **□** Vocational or technical school  **□** Other – please specify ________________________ |
| 4.5.3 | If no, how far is the nearest school from the village centre? | _______________________ km |
